# Hereditary angioedema with C1 inhibitor (C1-INH) deficit: the strength of recognition (51 cases)

**DOI:** 10.1590/1414-431X20187813

**Published:** 2018-11-14

**Authors:** N.T.M.L. Fragnan, A.L.N. Tolentino, G.B. Borba, A.C. Oliveira, J.A. Simões, S.M.U. Palma, R.N. Constantino-Silva, A.S. Grumach

**Affiliations:** 1 Pós-Graduação em Ciências da Saúde, Faculdade de Medicina do ABC, Santo André, SP, Brasil; 2 Curso de Graduação, Faculdade de Medicina do ABC, Santo André, SP, Brasil; 3 Departamento de Pediatria, Faculdade de Medicina do ABC, Santo André, Brasil; 4 Laboratório de Imunologia Clínica, Faculdade de Medicina do ABC, Santo André, SP, Brasil; 5 Disciplina de Imunologia Clínica, Faculdade de Medicina do ABC, Santo André, SP, Brasil

**Keywords:** Hereditary angioedema, C1 esterase inhibitor, Diagnosis, Treatment, Complement

## Abstract

Hereditary angioedema (HAE) is a rare autosomal dominant disease due to C1 esterase inhibitor deficiency (C1-INH). The disease is characterized by subcutaneous and submucosal edema in the absence of urticaria due to the accumulation of bradykinin. This descriptive study aimed to evaluate the clinical characteristics of patients with a confirmed diagnosis of HAE referred to our Outpatient Clinic between December 2009 and November 2017. Fifty-one patients (38 F, 13 M) with a mean age of 32 years (range: 7–70 y) were included. Family history of HAE was reported in 70% (36/51) of the cases; 33/46 patients became symptomatic by 18 years of age. The median time between onset of symptoms and diagnosis was 13 years (3 mo–50 y). The most frequent triggering factors for attacks were stress (74.4%), trauma (56.4%), and hormonal variations (56%). The main symptoms were subcutaneous edema in 93.5% (43/46) of patients, gastrointestinal symptoms in 84.8% (39/46), and obstruction in the upper airways in 34.8% (16/46). Hospitalization occurred in 65.2%, of whom 13.3% had to be transferred to the Intensive Care Unit. Prophylactic treatment was instituted in 87% (40/46) of patients, and 56.5% (26/46) required additional treatment to control attacks. Owing to our data collection over a period of 8 years, a significant number of patients were identified by this HAE reference center. Despite early recognition and prophylactic treatment, a high percentage of patients were hospitalized. HAE is still diagnosed late, reinforcing the need for more reference centers specialized in diagnosis and educational projects for health professionals.

## Introduction

Hereditary angioedema (HAE) is a rare disease with an autosomal dominant inheritance and an estimated prevalence of 1:50,000 (1). HAE results from deficit in the concentration and/or activity of the C1 esterase inhibitor (C1-INH), a protein belonging to the serpin family (2). This deficiency results in loss of the inhibitory function of the complement, fibrinolytic, coagulation, and contact systems (3). The contact system contributes most to the symptomatology of the disease, and the lack of control of this system generates excess bradykinin, a protein responsible for extravasation of plasma and consequently edema, as well as the contraction of smooth muscles, resulting in pain intensification (2,3). More than 400 different mutations have already been described in the C1-INH gene (*SERPING*1) (4).

HAE is still underdiagnosed by many health professionals. Patients face long periods from the onset of symptoms to proper diagnosis and treatment, with repercussions on morbidity and mortality (5,6). Patients often require urgent care and hospitalization, and misdiagnosis can result in unnecessary surgeries and recurrent work breaks. Without adequate treatment, a mortality of 25 to 40% is estimated, mainly due to laryngeal edema and asphyxia (7).

Clinical manifestations can be triggered by physical exercise, extreme temperatures, infection, changes in hormonal levels, emotional stress, and trauma, including surgical and dental procedures (8 [Bibr B09]). The symptoms usually begin in the first or second decade of life and are characterized by subcutaneous and/or submucosal edema, affecting the face, extremities, genitalia, oropharynx, larynx, and digestive system. The attacks are self-limiting, persist for up to 72 h, have variable recurrence, are not associated with urticaria, and do not respond to antihistamines, corticosteroids, or epinephrine (2,8–10).

The complement component 4 (C4) level can be used as a screening test for HAE diagnosis due to activation of the complement system by quantitative and/or qualitative deficiency of C1-INH. Consumption of C4 occurs even when patients are not under angioedema attacks, although up to 5% of the patients may not have reduced C4 (4,8,11). Diagnosis is confirmed by quantitative determination of C1-INH in approximately 85% of the cases (HAE with quantitative C1-INH deficiency), and a functional evaluation of C1-INH is needed in 15% of patients (HAE with C1-INH dysfunction) (2,4).

Treatment is based on the individual evaluation of the patient regarding the necessity for long-term prophylaxis, which can consist of attenuated androgens, antifibrinolytic agents, and plasma-derived C1-INH concentrate (pdC1-INH). Short-term prophylaxis is indicated for these patients prior to high-risk events (such as dental/surgical procedures) and may consist of plasma-derived C1-INH concentrate (pdC1-INH), attenuated androgen, or fresh frozen plasma. On-demand treatment can be done with pdC1-INH, icatibant, or ecallantide; the latter is not available in Brazil (2,5,12–16).

Considering that many patients with HAE go undiagnosed and that there is only one published Brazilian survey (5) on HAE – in which 210 patients were evaluated – this study aimed to describe the experience of a referral center in the follow-up of patients with HAE and discuss their clinical and laboratory characteristics in order to create awareness toward diagnosis.

## Material and Methods

This is a descriptive study of patients attending the Specialized Outpatient Clinic at Faculdade de Medicina do ABC between December 2009 and November 2017. HAE diagnosis was established by clinical symptoms and quantitative and/or functional C1-INH deficiency. C4 and C1-INH dosages were performed by nephelometry and/or radial immunodiffusion. The functional activity of C1-INH was evaluated by a chromogenic assay (Technoclone®, USA). The present study was approved by the Research Ethics Committee (CAAE 51896015.0.1001.0082), and patients and/or guardians signed consent forms. The variables underwent descriptive analysis, and quantitative data were analyzed by measures of central tendency.

## Results

**Figure 1.**
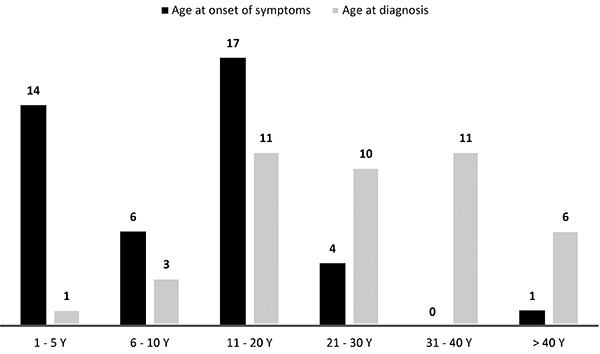
Age at the onset of symptoms and diagnosis of hereditary angioedema. Y: years.

Fifty-one patients with a confirmed HAE diagnosis and quantitative and functional C1 inhibitor deficiency (type I) (n=49; 96.1%) or only functional deficiency (type II) (n=2, 3.9%) were included. Of these, 38/51 (74.5%) were female, and 13/51 (25.5%) were males. They were aged between 7 and 70 years (mean: 34.5 y; median: 32 y), and 36/51 (70.6%) had a family history of suspected or confirmed hereditary angioedema. Symptoms were reported in 46/51 (90.2%), and 5/51 (9.8%) remained asymptomatic. In this study, 14/42 patients presented the first symptoms by the age of 5 years, 6/42 between 6 and 10 years, 17/42 between 11 and 20 years, and 5/42 older than 20 years (median: 11 years; range: 22–41 years). The time between onset of symptoms and diagnosis was up to 50 years (median: 13 years; range: 3 months to 50 years) (Figure 1).

The triggering factors reported were stress (29/39; 74.4%), trauma (22/39, 56.4%), hormonal variations associated with the menses, use of contraception and/or gestation (19/34 symptomatic women, 55.9%), and physical activity (3/39, 7.7%). One single triggering factor was reported by 5/39 (12.8%) patients and 2 or more by 34/39 (87.2%). Prodromes, up to 24 h before the attacks, were mentioned by 10/46 (21.7%) patients: paresthesia (n=5), erythema *marginatum* (n=3), and asthenia (n=2).

Regarding the area with edema, 43/46 (93.5%) reported subcutaneous and/or submucosal involvement, 39/46 (84.8%) had abdominal symptoms, and 16/46 (34.8%) had obstruction of the upper airways (Table 1). The initial frequency of symptomatic attacks was one or more per month in 28/41 (68.3%), one every 2–3 months in 4/41 (9.8%), one in 4–6 months in 8/41 (19.5%), and less than one attack/year in 1/41 (2.4%). A total of 30/46 (65.2%) of the patients required hospitalization during the attacks, of whom 4/30 (13.3%) were transferred to the intensive care unit at least once.

**Table 1.** Symptoms of patients with hereditary angioedema according to area of involvement.

	N	%
Asymptomatic	5/51	9.8
Symptoms	46/51	90.2
Skin		
Extremities	41/46	89.1
Face	21/46	45.7
Gastrointestinal		
Abdominal pain	38/46	82.6
Nausea/Vomiting	11/46	23.9
Distension/Edema	11/46	23.9
Diarrhea	4/46	8.7
Others		
Glottis	16/46	34.8
Vaginal	2/38*	5.3

*Symptomatic women.

During the diagnostic investigation, 10 (21.7%) patients underwent surgical procedures for symptoms that resembled HAE: appendectomy (n=6); exploratory laparotomy (n=2); cholecystectomy (n=1), and hysterectomy (n=1). Nine patients (19.6%) reported attacks following cesarean section (n=7), hepatic biopsy (n=1), and tonsillectomy (n=1).

C4 dosing was performed primarily or concomitantly with quantitative C1-INH dosing in 47/51 patients, and all patients presented values below the reference value (median=5.0 mg/dL, reference value: 1–40 mg/dL). The C1-INH levels among HAE patients with quantitative deficit were between 0.5 and 16 mg/dL (median=6.5 mg/dL, reference value: 21–40 mg/dL). The functional activity of C1-INH was assessed in 23 HAE patients with quantitative C1-INH deficiency, of whom 12 had levels below 50% (median: 28.9%; range: 3.4–48%), and 11 patients had normal values (median: 70%; range: 51–120%). The two patients with HAE with only functional deficit had activity levels of 0% and 18.4%.

Regarding treatment, 40/46 (87%) symptomatic patients underwent some type of continuous prophylactic treatment (Table 2). After the use of one type of continuous prophylactic medication, 23/40 (57.5%) achieved symptom control, of whom 15/23 (65.2%) initially had mild to moderate attacks. However, of the 17/40 patients that needed several prophylactic medications for attack management, 9 (52.9%) initially had severe attacks, and 11 (64.7%) had a frequency greater than 12 times per year. In 6/46 patients, no treatment was instituted: 4 presented only mild/moderate attacks, 1 presented a single severe attack during cesarean section, and 1 presented severe attacks but refused prophylactic treatment.

**Table 2.** Prophylactic treatment of patients with hereditary angioedema.

	% (n)	Dose (mg/day)
No treatment	13 (6/46)	
Monotherapy	57.5 (23/40)	
Tranexamic acid	34.8 (8/23)	250-1500
Oxandrolone	34.8 (8/23)	2.5-7.5
Danazol	26.1 (6/23)	100-200
Epsilon aminocaproic acid	4.3 (1/23)	500
Combination	42.5 (17/40)	
Tranexamic acid+oxandrolone	41.2 (7/17)	25-1500+2.5-7.5
Tranexamic acid+danazol	29.4 (5/17)	500-2250+100-400
Danazol+oxandrolone	23.5 (4/17)	100-600+2.5-7.5
Epsilon aminocaproic acid+oxandrolone	1/17	500+2

**Figure 2.**
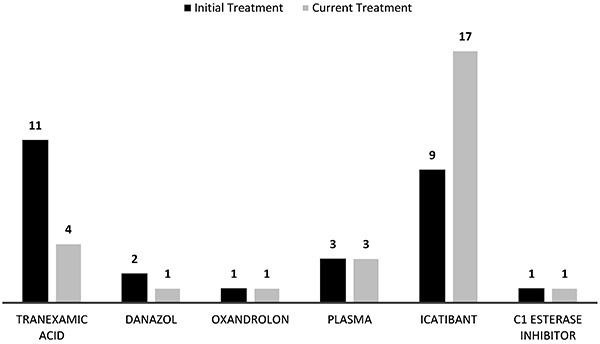
Profile of initial and current treatment of attacks in patients with hereditary angioedema.

After continuous prophylactic treatment, 15/40 (37.5%) patients did not present new attacks. Of the 25/40 patients (67.5%) who required medications for specific treatment during attacks, 5 were not able to access the appropriate therapy for this purpose, and had the dose of tranexamic acid, danazol, or oxandrolone increased during attacks. This practice was also established in 8/40 patients until the patient or a family member had access to medication. Twelve of 40 patients had already received an on-demand treatment after diagnosis such as icatibant, pdC1-INH, or fresh frozen plasma; 9 of these 12 patients had a family member who gave them access to drugs to treat attacks (Figure 2).

## Discussion

Considering the estimated prevalence of HAE of 1:50,000, the Brazilian population would have approximately 4,400 individuals affected, but the Brazilian HAE patients association (ABRANGHE) reported 1,235 patients in 2016. It should be noted that these data were recorded by patients and/or family members themselves and probably included other types of angioedema without urticaria (1).

The region served by the newly established HAE reference center comprises 2,500,000 inhabitants. Thus, we estimate that at least 50 patients with HAE would be in the region. However, the rapid increase in the population with HAE observed in approximately 8 years of work reflects the referral of patients from several regions of the country due to this center's status as a reference center. Thus, the high number of new cases in our region is expected, but the lack of disease recognition by a significant number of physicians, even among specialists, can make this action difficult (13).

Although HAE has an autosomal dominant inheritance, the disease is not equally distributed between males and females. There was a predominance of women affected, as previously described (5,6,17,18), which may be related to hormonal influence, mainly estrogenic, as a crisis trigger (8,19).

Family history was evidenced in approximately 2/3 of the patients, as described previously (13,20). The lack of affected relatives in approximately 25% of the cases may be associated with new mutations, although clinically these patients do not differ from those with familial inheritance (15). On the other hand, a report from Italy observed that 43% of the patients did not have a family history, although the authors emphasized that maybe the relatives had not yet been diagnosed (6). In our series, 5 patients remained asymptomatic and were identified because they had symptomatic relatives. We actively searched for HAE among family members, and the identification of asymptomatic patients corroborates the importance of early guiding and establishing treatment, if necessary.

In some cases, the low penetrance of the disease may explain why individuals with laboratory abnormalities and/or mutations do not present symptoms, as found in 5 (9.8%) of the patients evaluated here. It has been suggested that approximately 5% of HAE patients are asymptomatic, a percentage lower than what we found (1,21).

Symptoms usually begin early in childhood, and approximately half of our patients developed the first symptoms within 10 years of age (8). Approximately 90% of our patients were symptomatic by age 20, higher than what was observed by other authors (1,8,22). Among women, 24/32 had symptoms at or before the age of 16, reinforcing the possible correlation between symptom onset and the oscillations that occur during puberty and the use of oral contraceptives (2,8).

Although the first clinical manifestations occur in childhood, most patients are diagnosed only after several years (17,21,23). Lunn et al. (20) evaluated 313 patients from several European countries and the United States. The mean time from onset to diagnosis was 8.3 years, and these patients were previously evaluated by 4.4 physicians on average. We found an average of 13 years from symptom onset to HAE identification. It is noteworthy that 10 patients had symptoms for more than 30 years without having a correct diagnosis, of whom 6 had a family history and were identified only after younger relatives sought specialized medical care.

Recurrent episodes of edema not associated with urticaria involving the skin and submucosa of various organs characterize HAE. The frequencies of both skin and gastrointestinal tract edema that we observed were similar to those of other reports (1,8,21). The main factor associated with mortality in HAE is upper airway edema, which according to Bork et al. (8), represents 1 in 125 cases of edema in patients with HAE. In our series, 1/3 of the patients reported edema in the airways. Bygum (21) reported that 54.5% of the 82 patients evaluated in Denmark had laryngeal edema, and in 20.8%, this edema was confirmed.

It is not uncommon for patients in HAE attacks to undergo unnecessary surgeries (7,24). Eleven (23.9%) of our patients had undergone surgical procedures due to errors in diagnosis during angioedema crises, a number similar to that described in developed regions such as the United States (19%) and Europe (24%). One mistake that stands out was the hysterectomy in a patient due to abdominal pain associated with gynecological symptoms.

Thirty-nine symptomatic patients reported that attacks were triggered by a factor; stress was the most cited, followed by traumas, hormonal variations, and surgical procedures, similar to other studies (25). In many attacks, the factor was not identified, which reinforces the assertion that attacks may also be spontaneous (1,25).

Some patients reported signs and symptoms that preceded attacks, usually within 24 h: erythema serpiginous, irritability, weakness, nausea, and asthenia are the most prevalent in the literature (2,5,13). In this study, only 9/46 patients reported prodromes, much lower than the number described by Reshef et al. (26), in which 82.5 to 95.7% of the patients (depending on the questionnaire applied) recognized a prodrome. It is possible that the low incidence of prodromes was due to lack of questioning during follow-up.

The C4 dosage is a good screening test for diagnosis in patients suspected of HAE. All of our patients had decreased C4 values, but other studies have found normal C4 concentrations in 2 to 5% of cases (8,11). This information is relevant due to the restricted access to the more specialized diagnostic exams in our country (5). To confirm HAE, quantitative dosing of C1-INH should be performed. The functional defect of C1-INH was identified in a smaller proportion of our population compared to that of other studies (1,6,22). The limited access to functional C1-INH could have influenced this finding (5).

The treatment of HAE is based on long-term prophylaxis, short-term prophylaxis before high risk procedures, and the treatment of crises (on demand) (12,27). In our population, 87% of the symptomatic patients underwent or had already undergone some type of prophylactic treatment. Current recommendations reinforce that the need for long-term prophylaxis should be reassessed at each patient's visit, prioritizing on-demand treatment, due to less side effects and better quality of life of patients with on-demand treatment (12). The high frequency of patients undergoing prophylactic treatment corroborates the difficulty in treating attacks in Brazil (12,13,28).

In our group, monotherapy was effective for 53% of women and 64% of men. However, in 42% of cases, a combination of drugs was necessary, opting mainly for an antifibrinolytic agent and an attenuated androgen (only 4 men used attenuated androgens in combination). This was done because of the inadequate control of attacks and difficulty of accessing first-line drugs for control, as well as to avoid significant increases in androgen doses, preventing side effects (2,23). Prophylactic treatment with plasma-derived or recombinant C1-INH is not available in our country.

Attenuated androgens (danazol, stanozolol, and oxandrolone) are considered effective drugs for long-term prophylaxis because they result in an increase in C1-INH and C4 levels reducing the frequency of attacks. According to the Gargnano Consensus (29), the dose of danazol is limited to 200 mg per day to reduce the incidence of side effects. Zuraw et al. (3) observed that up to 30% of patients may not achieve control of attacks with the use of these drugs, and a significant number discontinue use due to the intensity of side effects. Despite this shortcoming and the absolute contraindication in gestation and relative contraindication in childhood, danazol remains the only drug advocated for long-term prophylaxis made available by the Brazilian government (2,14,30,31 [Bibr B32]). The normalization of the C1-INH level by danazol explains the normal dosages of C1-INH (16 mg/dL) at the initial evaluation in our outpatient clinic when it was already used by the patient.

Antifibrinolytic agents (epsilon-aminocaproic acid and tranexamic acid) are considered effective in preventing HAE attacks in one-third of patients (2,31–33). In patients in whom tranexamic acid was effective in the control of attacks, doses did not exceed 1,500 mg/day. In patients who required a higher dose, additional medication was added to control the disease.

Most of our patients were hospitalized during the attacks, of whom 13.3% were transferred to the ICU at least once because of the intensity of the condition or mainly due to the absence of diagnosis and, especially, adequate treatment. In European countries, Aygören-Pürsün et al. (17) reported that 91% of the patients had specific treatment available at home, and 23% sought emergency care due to more intense attacks than usual, but only 14% were hospitalized. This result demonstrates the inaccessibility to on-demand treatment in Brazil, or even a possible lack of adherence to it (unpublished data).

Of our patients, despite the difficulty of acquiring pdC1-INH as continuous prophylaxis, three of them used it, and two of them used it every 7 to 10 days. This form of administration is not recommended, but the fear of being left untreated has caused patients to use this dosage. One patient used pdC1-INH for only 3 months because of the inconvenience of the intravenous application. Although it is considered safe and effective, intravenous administration of pdC1-INH has been associated with venous thrombosis, probably due to the fully implanted catheter (34). More recently, the use of subcutaneous pdC1-INH for prophylaxis of HAE has been published, but it is not yet available in our country (35).

In our series, 9 patients reported attacks following invasive procedures and most of them still did not have adequate diagnosis and treatment of the disease. The procedures that confer the greatest risk for patients with HAE are dental extraction, tonsillectomy, facial surgery, endoscopy, and bronchoscopy; in short, surgical procedures that require tracheal intubation (17). Seven patients reported attacks during or after delivery, all of them cesarean sections. However, the type of delivery was not related to a higher risk of attacks in another study (36).

Of the symptomatic patients in follow-up, 25/46 still presented attacks, despite the daily use of prophylactic medication, requiring medications for immediate treatment. Four patients took fresh frozen plasma, with improvement of symptoms after a few hours and no side effects. However, there is no controlled study supporting its role or efficacy, and it can worsen the condition, since plasma also contains the substrate of the inhibitor (37). Icatibant, a competitive and selective antagonist of the bradykinin B2 receptor, was used by most of our patients to treat attacks. This drug was the first to be available in our country for the treatment of attacks (2,27). The pdC1-INH concentrate was used as a treatment in crisis only by one patient of this sample, mainly due to the recent introduction of this medication in the country and its difficult access. As an alternative treatment, several patients increased the doses of continuous medications (danazol, tranexamic acid, and oxandrolone) to control attacks. This approach is recommended against in all consensus and guidelines (2,31,38).

HAE is still a disease barely known to both the general population and the vast majority of medical staff, which is demonstrated by the long interval between the onset of symptoms and the diagnostic confirmation. This highlights the necessity for more referral centers that can perform the diagnosis as well as the education and training of professionals in the recognition and correct routing of suspected cases. HAE is a disease with high morbidity and mortality, with several patients remaining without adequate prophylactic or on-demand treatment, incurring a huge cost to the health system due to the high rate of absences from work, inability to perform basic self-care tasks, and, mainly, the high risk of mortality. The resources to identify the patients are not difficult to access or costly, such as the dosage of C4.

Therefore, the awareness of the clinical aspects of HAE and the ability to differentiate it from allergic manifestations and other diseases are essential for the early diagnosis and therapy, improving the quality of life of HAE patients.
